# Sotolon and (2*E*,4*E*,6*Z*)-Nona-2,4,6-trienal Are the Key Compounds in the Aroma of Walnuts

**DOI:** 10.1021/acs.jafc.3c01002

**Published:** 2023-04-26

**Authors:** Christine A. Stübner, Martin Steinhaus

**Affiliations:** Leibniz Institute for Food Systems Biology at the Technical University of Munich (Leibniz-LSB@TUM), Lise-Meitner-Straße 34, Freising 85354, Germany

**Keywords:** walnut, Juglans regia L., sotolon, (2*E*,4*E*,6*Z*)-nona-2,4,6-trienal, aroma extract dilution analysis (AEDA), stable isotopically substituted odorants, odor activity value (OAV), aroma reconstitution

## Abstract

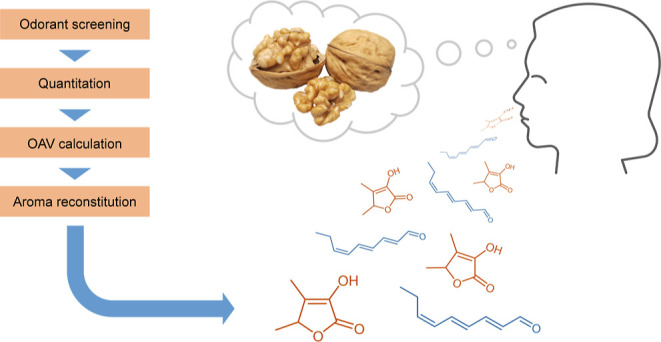

Fresh kernels of the walnut tree (*Juglans regia* L.) show a characteristic and pleasant aroma, the molecular basis of which was unknown. The application of an aroma extract dilution analysis resulted in 50 odor-active compounds. Among them, 37 had not been reported as fresh walnut kernel volatiles before, including the two odorants with the highest flavor dilution factors, namely, fenugreek-like smelling 3-hydroxy-4,5-dimethylfuran-2(5*H*)-one (sotolon) and oatmeal-like smelling (2*E*,4*E*,6*Z*)-nona-2,4,6-trienal. Quantitations revealed 17 odorants with concentrations in the walnuts that exceeded their odor threshold concentrations. Aroma reconstitution and omission experiments finally showed that the characteristic aroma of fresh walnuts is best represented by a binary mixture of sotolon and (2*E*,4*E*,6*Z*)-nona-2,4,6-trienal. Of both, the natural concentration was ∼10 μg/kg. Further sensory studies showed that the walnut character is intensified when their concentrations are in parallel increased to ∼100 μg/kg. This finding may guide the future breeding of new walnut cultivars with improved aroma.

## Introduction

The walnut tree (*Juglans regia* L.) is a huge tree with heights up to 30 m. It is native to a region in Eurasia stretching from southern Europe and the Near East to the Himalayan region and China.^1^ Cultivation started more than 2000 years ago.^2^ Today, walnut trees are grown worldwide in temperate and subtropical climates, predominantly for nuts. Fruits do not develop before an age of 15–20 years. The fruits are surrounded by a green fleshy husk and consist of a brown, woody, bipartite pericarp and a single edible seed with a light brown seed coat and huge wrinkled cotyledons.^1^ The seeds are high in fat and fiber and commonly referred to as walnut kernels. Major exporting countries of whole and shelled walnuts are currently China, the USA, Iran, and Turkey.^3^ Raw or toasted, walnut kernels are a popular snack and a common ingredient in bakery products and sweets, and also widely used as a garnish.

Fresh walnut kernels are particularly valued for their characteristic aroma, which is clearly different from that of other tree nuts such as almonds, cashew nuts, and hazelnuts. The first researchers interested in the molecular background of walnut aroma were Clark and Nursten in 1976.^4^ They isolated walnut volatiles from the extracted oil in two different ways—one based on Likens-Nickerson extraction, the other one based on a milder, artifact-avoiding high vacuum degassing approach. The isolates were analyzed by gas chromatography–mass spectrometry (GC–MS) and gas chromatography–olfactometry (GC–O) using columns of different polarity. Up to 103 peaks could be separated in the chromatograms, however, none of them showed a specific walnut-like odor. Clark and Nursten concluded that the “odor of walnuts appears to be due to the collective effect of a number of components”.^4^ This assumption was confirmed in a subsequent study by the same authors.^5^ This time, walnut volatiles were directly sampled from the headspace above the kernels. Again, GC–O and GC–MS analyses of the trapped volatiles did not reveal any peak with a specific walnut odor. However, when the entire eluate of the GC column was collected, its odor was clearly walnut-like. Fractionation experiments indicated that carbonyl compounds contributed to the walnut-like odor whereas alcohols did not. Which individual compounds play the key role in walnut aroma, however, remained unclear. Different mixtures of major carbonyl compounds among which were hexanal, pentane-2,3-dione, 2-methylpent-2-enal, and pentanal resulted at best only in a moderately walnut-like aroma.

For many years, the topic of the molecular background of the characteristic walnut aroma was not pursued further. Instead, research on walnut volatiles was focused on differences between origins,^6^ differences between varieties,^7^ their antioxidant potential,^8^ and their suitability to assess the oxidative stability of walnuts after processing and storage.^7,9,10^

More recently, Liu et al.^11^ attempted to reinvestigate the compounds responsible for the aroma of walnuts. They isolated the volatiles from raw and roasted walnut kernels by solvent-assisted flavor evaporation (SAFE)^12^ and screened them for odorants by GC–O in combination with aroma extract dilution analysis^13^ (AEDA). In the raw walnuts, 29 odor-active compounds were detected in a flavor dilution (FD) factor range of 1 to 243, among which 11 showing FD factors ≥9 were quantitated and 10 finally resulted in concentrations beyond the odor threshold concentration (OTC) corresponding to odor activity values (OAV = concentration in walnut/OTC) of ≥1. The highest OAVs were obtained for some fat oxidation products such as (2*E*)-non-2-enal (OAV 2217), octanal (OAV 769), hexanal (OAV 753), and nonanal (OAV 500). With this result, Liu et al. declared that they had “provided the integral determination of the key aroma-active compounds” in raw walnuts. However, they did not provide proof of their statement through an aroma reconstitution experiment.^14^ When we prepared a solution of the 10 compounds proposed by Liu et al. as key odorants in raw walnuts in the reported concentrations and with an odorless mixture of medium-chain triglycerides as the solvent in our lab, we found that it showed an intense fatty and rancid odor but lacked the specific walnut-like character.

The objectives of the current study were therefore to re-screen the volatiles isolated from raw walnuts for odor-active compounds with a focus on potent odorants that had been overlooked in the previous studies, determine their natural concentrations, and assess their role in the overall raw walnut kernel aroma not only based on OAV calculations but eventually also by aroma reconstitution and omission experiments^14,15^ with the aim to unequivocally identify the compounds responsible for the walnut character.

## Materials and Methods

### Nuts

All nut kernels used in this study were purchased at the local retail market in Freising, Germany. In all cases, the kernels were dried but unroasted and the labeling indicated that they had been packaged under an inert gas atmosphere. The walnut sample was selected from numerous brands based on its characteristic and pronounced walnut aroma and the absence of rancid and other off-flavors, which easily develop when walnut kernels are stored in the presence of oxygen. All analyses were performed immediately after opening the package, in most cases within 2 days after purchase or at least before the best-before date.

### Reference Odorants

The following compounds were purchased from commercial sources: **1**–**4**, **6**, **8**–**13**, **15**–**19**, **20**–**26**, **28**–**31**, **34**, **39**–**42**, **44**–**50** (Merck; Darmstadt, Germany), **5**, **36**, **37**, **43** (Thermo Fisher Scientific; Waltham, MA, USA), **7**, **32** (Toronto Research Chemicals; Toronto, Canada), **35** (Carl Roth; Karlsruhe, Germany), and **38** (Cayman Chemicals Company; Ann Arbor, MI, USA). Compound **14** was synthesized according to a procedure described in the literature.^16^ Compound **21** was freshly distilled before use. Compound **27** was obtained from a commercial sample of **28** as detailed earlier.^17^ The same approach was used to prepare compound **20** from a commercial sample of **24**. Compound **33** was synthesized according to Schuh and Schieberle^18^ and underwent a first purification step by column chromatography as detailed in their paper. A second and final purification step was performed by preparative HPLC using a system from Knauer (Berlin, Germany) equipped with an Azura sampler AS 6.1 L, an Azura pump P6.1L HPG, an Azura detector MWD 2.1L, and a fraction collector LABOCOL Vario 4000. The column was a Eurosphere II Diol 100-5 (250 × 8 mm). The injection volume was 100 μL and the flow rate was 1.6 mL/min. Solvent A was *n*-hexane/ethanol 90/10 and solvent B was *n*-hexane/ethanol 70/30. The separation program was 0–20 min A/B from 100/0 to 90/10, 20–23 min to 0/100, 23–26 min back to 100/0, and continued 26–30 min. Ultraviolet detection was performed at 220 nm. For data evaluation, the Purity Chrome software, version 5.09.069 was used.

### Stable Isotopically Substituted Odorants

The compounds (^2^H_3_)-**9**, (^13^C_2_)-**10**, and (^13^C_2_)-**49** were purchased from Merck. Compound (^2^H_3_)-**30** was from Cambridge Isotope Laboratories (Tewksbury, MA, USA). (^2^H_2_)-**5**, (^2^H_2_)-**7**, (^2^H_2_)-**8**, (^13^C_2_)-**12**, (^2^H_3_)-**13**, (^2^H_2_)-**14**, (^2^H_2_)-**15**, (^2^H_2_)-**17**, (^2^H_2_)-**19**, (^2^H_2_)-**22**, (^13^C_2_)-**24**, (^13^C_2_)-**28**, (^2^H_3_)-**31**, (^13^C_2_)-**33**, (^2^H_2_)-**34**, (^2^H_2_)-**38**, (^13^C_2_)-**40**, (^13^C_2_)-**46**, (^2^H_3_)-**47**, and (^2^H_3_)-**50** were synthesized according to procedures detailed in the literature; individual references are available in the Supporting Information, Table S1. Compound (^13^C_2_)-**33** was purified as detailed above for the isotopically unmodified compound **33**.

### Miscellaneous Chemicals

Diethyl ether was purchased from CLN (Freising, Germany) and was freshly distilled through a column (120 cm × 5 cm) packed with Raschig rings before use. Odorless silicone oil was from Merck. Medium-chain triglycerides, type Miglyol 812, and silica gel 60 (0.040–0.63 mm) were purchased from VWR (Darmstadt, Germany). The silica gel was purified as detailed previously.^19^

### Gas Chromatography

GC–O analyses were performed with a GC–O/FID instrument. For GC–MS analyses, four different instruments were used: a one-dimensional GC–MS instrument with a Paul trap mass analyzer, a two-dimensional heart-cut GC–GC–MS instrument with a Paul trap mass analyzer, a two-dimensional heart-cut GC–GC–HRMS instrument with an orbitrap mass analyzer, and a comprehensive two-dimensional GC×GC–MS instrument with a time-of-flight mass analyzer. Details on the individual instruments are available in the Supporting Information.

### Aroma Extract Dilution Analysis

Walnut kernels (150 g) were crushed down to a particle size of ∼1–3 mm using a mortar and pestle. A portion (50 g) of the crushed kernels was placed in a 2 L amber-colored wide-neck Erlenmeyer flask. Under ice-cooling, saturated calcium chloride solution was added (100 mL) to stop enzymatic reactions,^20^ before the mixture was homogenized with a stainless-steel blender to facilitate the following extraction step. Diethyl ether (350 mL) was added and the mixture was stirred at ambient temperature in the dark overnight. Under ice cooling, anhydrous sodium sulfate (300 g) was added and the organic phase was decanted through a folded filter paper. The residue was washed with diethyl ether (3 × 100 mL) and the organic phases were combined. Nonvolatiles were removed by automated solvent-assisted flavor evaporation (aSAFE)^21^ at 40 °C using an open/closed time combination of the pneumatic valve of 0.1 s/10 s. The distillate was dried over anhydrous sodium sulfate (50 g) and concentrated to a volume of 0.5 mL, first using a Vigreux column (50 × 1 cm) and finally a Bemelmans microdistillation device.^22^ When a drop of this volatile isolate was placed on a fragrance test strip and the odor was evaluated directly after evaporation of the solvent, the characteristic walnut aroma was clearly perceivable.

The walnut volatile isolate was subjected to GC–O analysis using the GC–O/FID instrument detailed in the Supporting Information with the FFAP column. Two trained and experienced assessors with complementary olfactory capabilities^14^ repeatedly performed GC–O until results were reproducible. By stepwise 1:2 dilution of the volatile isolate with diethyl ether, dilutions of 1:2, 1:4, 1:8, 1:16, 1:32, 1:64, 1:128, 1:256, 1:512, 1:1024, and 1:2048 of the initial solution were prepared and subjected to GC–O analysis. Each odor-active compound was assigned an FD factor corresponding to the dilution factor of the highest diluted sample in which the odor was perceived by any of the two assessors.^14^

Toward structural identification of the odor-active compounds, odor description and retention index (RI) on the FFAP column were first compared with data compiled in databases.^23,24^ Structure proposals were verified by GC–O of authentic reference compounds. If this verification was successful, further confirmation was sought by parallel GC–O analysis of the walnut volatile isolate and the reference compounds using the DB-5 column. Final structure confirmation was achieved by comparing mass spectra of the compounds in the walnut volatile isolate with mass spectra of the reference compounds analyzed under identical conditions. To minimize coelution problems, the GC×GC–MS instrument was employed for this purpose.

### Odorant Quantitation

The workup of the walnut kernels (50–150 g) was performed as detailed in the AEDA section. The stable isotopically substituted odorants used as internal standards (cf. Supporting Information, Table S2) were added to the first diethyl ether portion in the extraction step. Depending on the expected target compound concentrations, amounts of the added internal standards ranged from 0.06 to 14.7 μg. The aSAFE distillates were concentrated to a volume of 100 μL and subjected to GC–MS analysis using the heart-cut GC–GC–MS instrument (**5**, **8**, **14**, **15**, **19**, **22**, **23**, and **30**), the heart-cut GC–GC–HRMS instrument in the positive CI mode (**17** and **34**), the heart-cut GC–GC–HRMS instrument in the negative CI mode (**38**), or the GC×GC–MS instrument (**7**, **9**, **10**, **12**, **13**, **20**, **24**, **27**, **28**, **31**, **32**, **40**, **46**, **47**, **49**, and **50**). All quantitations were performed in duplicates or triplicates.

Peak areas corresponding to the analyte and internal standard were obtained from the extracted ion chromatograms using characteristic quantifier ions. Odorant concentrations in the walnut kernels were calculated from the area counts of the analyte peak, the area counts of the standard peak, the amount of walnut used for the workup, and the amount of standard added, by employing a calibration line equation. The calibration line equation was obtained by linear regression after analysis of analyte/standard mixtures in different concentration ratios. Quantifier ions and calibration line equations are available in the Supporting Information, Table S2. Individual concentration data and standard deviations are available in the Supporting Information, Table S3.

### Odor Threshold Concentrations

These were determined orthonasally in low-odor sunflower oil according to the American Society for Testing and Materials standard practice for determination of odor and taste thresholds by a forced-choice ascending concentration series method of limits.^25^ Test compounds were checked for purity by AEDA before use and considered suitable for the OTC determination if the FD factor of the target compound was at least 100 times higher than the FD factor of the most potent impurity. Spiked samples were prepared by adding the test substance in ethanolic solution to the oil. To the reference samples, a corresponding amount of pure ethanol was added. The final ethanol concentrations were kept below 300 μL/kg oil. Between two consecutive three-alternative forced choice tests, odorant concentrations differed by a factor of 3. Samples (20 g) were presented to the assessors in cylindrical single-use polystyrene vessels (40 mL nominal volume) with polytetrafluoroethylene lids. The tests were carried out at 22 ± 2 °C room temperature by 12–20 trained assessors in separate booths of a room exclusively dedicated to sensory evaluations.

### Sensory Evaluation of Walnut Aroma Model Mixtures

The general matrix used for the aroma reconstitution experiments, omission experiments, and sensory tests with different sotolon/(2*E*,4*E*,6*Z*)-nona-2,4,6-trienal mixtures was an emulsion obtained by mixing 29 g of odorless silicone oil with 1 g of an aqueous phase buffered to a pH of 6.5 (H_2_PO_4_^–^/HPO_4_^2–^). The pH corresponded to the pH measured in a homogenate of the walnut kernels with a minimum amount of demineralized water. Aliquots of ethanolic or aqueous stock solutions of the reference odorants were added either to the silicone oil or to the aqueous phase before mixing. Final ethanol concentrations were kept below 300 μL/kg. The model mixtures (30 g) were presented in 100 mL Erlenmeyer flasks with glass stoppers under magnetic stirring to a panel of 14–18 trained assessors. The tests were carried out at 22 ± 2 °C room temperature in the room described before. Assessors were asked to orthonasally rate the intensities of descriptors defined by reference materials. The descriptors “fenugreek” and “oatmeal” were defined by aqueous solutions of sotolon and (2*E*,4*E*,6*Z*)-nona-2,4,6-trienal, respectively. Concentrations were 100 times the OTC. The descriptor “walnut” was defined by freshly crushed walnut kernels. Ratings of all panelists were averaged by calculating the arithmetic mean.

## Results and Discussion

### Odorant Screening

GC–O in combination with AEDA applied to the volatile isolate obtained from walnut kernels with a characteristic aroma profile resulted in 50 odor-active compounds, all of which were successfully identified (Table 1). Surprisingly, only 13 of the 50 compounds had previously been reported in walnuts (Table 1, rightmost column). Among the other 37 compounds, 12 were known as walnut oil volatiles, but 25 were unknown in walnuts as well as in walnut oil.

**Table 1:** Odorants in the Volatile Isolate Obtained from Walnut Kernels

no.	odorant^a^	odor^b^	RI^c^ FFAP	RI^c^ DB-5	FD factor^d^	previously reported^e^
1	butane-2,3-dione	buttery	982	603	2	5/42
2	hexanal	green, grassy	1080	802	2	5/43
3	γ-terpinene	earthy	1234	1059	4	–/44
4	octanal	citrusy	1285	1005	4	6/45
5	oct-1-en-3-one	mushroom	1293	979	256	6/37
6	2-ethylpyrazine	roasty	1331	916	8	–/38
7	(5*Z*)-octa-1,5-dien-3-one	geranium leaf	1364	982	16	–/–
8	(2*E*)-oct-2-enal	fatty, citrusy	1419	1061	64	6/38
9	3-isopropyl-2-methoxypyrazine	bell pepper	1417	1086	64	–/–
10	acetic acid	vinegar	1450	636	16	46/38
11	methional	cooked potato	1455	910	4	–/–
12	(2*E*,4E)-hepta-2,4-dienal	floral, fatty	1480	1015	16	6/47
13	3-*sec*-butyl-2-methoxypyrazine	bell pepper	1496	1167	64	–/–
14	(2*Z*)-non-2-enal	fatty, floral	1494	1148	32	–/42
15	(2*E*)-non-2-enal	cucumber, green	1532	1163	16	6/38
16	2-methylpropanoic acid	sweaty, cheesy	1560	783	8	–/42
17	(2*E*,6*Z*)-nona-2,6-dienal	cucumber, green	1584	1154	32	–/–
18	undecanal	fatty, floral	1600	1306	8	–/–
19	butanoic acid	sweaty, cheesy	1627	827	16	48/38
20	(2*E*,4*Z*)-nona-2,4-dienal	fatty	1639	1197	16	–/–
21	phenylacetaldehyde	floral, honey	1639	1047	8	48/38
22	3-methylbutanoic acid	sweaty, cheesy	1667	863	16	–/44
23	2-methylbutanoic acid	sweaty, cheesy	1668	857	16	–/–
24	(2*E*,4*E*)-nona-2,4-dienal	fatty	1692	1215	32	–/38
25	(2*E*)-undec-2-enal	green, soapy	1747	1362	8	–/49
26	α-farnesene	green	1745	1509	8	–/–
27	(2*E*,4*Z*)-deca-2,4-dienal	fatty, deep-fried	1752	1296	32	–/42
28	(2*E*,4*E*)-deca-2,4-dienal	fatty, deep-fried	1808	1322	32	11/43
29	cyclotene	fenugreek	1819	1024	8	–/42
30	hexanoic acid	sweaty, cheesy	1838	1015	16	50/38
31	2-methoxyphenol	smoky	1862	1087	256	–/–
32	(2*E*,4*E*,6*Z*)-nona-2,4,6-trienal	oatmeal	1876	1273	1024	–/–
33	(2*E*,4*E*,6*E*)-nona-2,4,6-trienal	oatmeal	1895	1285	2	–/–
34	γ-octalactone	coconut	1918	1255	32	–/–
35	β-ionone	floral, raspberry	1928	1480	4	–/42
36	δ-octalactone	coconut	1967	1292	4	–/–
37	maltol	caramel	1974	1114	4	–/38
38	*trans*-4,5-epoxy-(2*E*)-dec-2-enal	metallic	2004	1382	256	–/–
39	4-methoxybenzaldehyde	aniseed, woodruff	2031	1259	8	–/–
40	HDMF^f^	caramel	2033	1087	256	–/38
41	EHMF^g^	caramel	2077	1139/1148^h^	8	–/–
42	4-hydroxy-5-methylfuran-3-one	fruity, caramel	2127	1065	4	–/–
43	γ-decalactone	coconut	2133	1496	4	–/–
44	eugenol	clove	2169	1354	8	11/–
45	(2*Z*,4*Z*)-δ-deca-2,4-dienolactone	sweet, coconut	2170	1459	8	–/–
46	sotolon	fenugreek	2205	1111	512	–/–
47	2′-aminoacetophenone	foxy	2222	1304	64	–/–
48	(6*Z*)-γ-dodec-6-enolactone	sweet, fruity	2389	1658	4	–/–
49	2-phenylacetic acid	floral, honey	2553	1267	64	–/–
50	vanillin	vanilla	2573	1400	64	–/–

a Each odorant was identified by comparing its retention indices on two GC columns of different polarity (DB-FFAP and DB-5), its mass spectrum obtained by GC–MS, as well as its odor as perceived at the sniffing port during GC–O to data obtained from authentic reference compounds analyzed in parallel.

b Odor as perceived at the sniffing port during GC–O.

c Retention index; calculated from the retention time of the compound and the retention times of adjacent *n*-alkanes by linear interpolation.

d Flavor dilution factor; dilution factor of the highest diluted walnut volatile isolate in which the odorant was detected during GC–O analysis by any of two assessors.

e References that first reported the compound as fresh walnut kernel volatile/walnut oil volatile; the minus sign (−) indicates that there was no report in the literature yet.

f 4-Hydroxy-2,5-dimethylfuran-3(2*H*)-one.

g 2-Ethyl-4-hydroxy-5-methylfuran-3-one.

h EHMF is separated from its tautomer 5-ethyl-4-hydroxy-2-methylfuran-3-one on the DB-5 column, on the DB-FFAP column no separation of the isomers was observed.

The odor descriptions were highly diverse. Frequently mentioned descriptors included fatty (8×), floral (6×), green (5×), and sweaty, cheesy (5×). None of the odorants was described as specifically walnut-like. This confirmed earlier results^4,5,11^ and supported the hypothesis of Clark and Nursten^4,5^ that walnut aroma is formed by a combination of compounds and is not caused by a single odorant.

FD factors ranged from 2 to 1024. The compounds with the highest FD factors were oatmeal-like smelling (2*E*,4*E*,6*Z*)-nona-2,4,6-trienal (**32**; FD factor 1024) and fenugreek-like smelling 3-hydroxy-4,5-dimethylfuran-2(5*H*)-one, better known as sotolon (**46**; FD factor 512). Both compounds had not been reported as walnut constituents before. (2*E*,4*E*,6*Z*)-Nona-2,4,6-trienal is the character impact compound in the aroma of oatmeal,^18^ it substantially contributes to the aroma of black tea,^26^ and it has been reported as an odor-active compound in a variety of other foods such as green tea,^27^ hog plum pulp,^28^ and prawns.^29^ (2*E*,4*E*,6*Z*)-Nona-2,4,6-trienal is formed from linolenic acid.^18^

Sotolon is the character impact compound in many herbs, spices, and seasonings used to flavor savory foods. Herbs and spices include fenugreek seeds, fenugreek leaves, lovage leaves, Transcaucasian hogweed shoots, and blue melilot shoots.^30−32^ Ground fenugreek seeds are widely used in commercial curry powders. For this reason, the odor of sotolon is also often described as curry (powder)-like. Fenugreek leaves are used in Indian curry dishes. Fresh lovage leaves and dried Transcaucasian hogweed shoots are used to season soups. Whereas lovage leaves are used all over Europe, Transcaucasian hogweed is specifically used in Armenia to flavor Karshm soup, a local specialty.^31^ Dried blue melilot shoots are used in the European alpine region to season local bread and cheese types. Not least, sotolon substantially contributes to the characteristic aroma of soy sauce.^33^ Sotolon is not only biochemically formed but also during thermal food processing in the course of the Maillard reaction,^34^ for example during pan frying of white mushrooms.^35^ Recent metaanalysis has identified sotolon as one of the generalists among the odorants in food, that is, it shows an exceptionally great abundance.^36^

In the order of decreasing FD factors, (2*E*,4*E*,6*Z*)-nona-2,4,6-trienal and sotolon were followed by a group of four compounds, all of which showed an FD factor of 256. These four compounds were mushroom-like smelling oct-1-en-3-one (**5**), caramel-like smelling 4-hydroxy-2,5-dimethylfuran-3(2*H*)-one (HDMF; **40**), also known by its trade name Furaneol, metallic smelling *trans*-4,5-epoxy-(2*E*)-dec-2-enal (**38**), and smoky smelling 2-methoxyphenol (**31**). Oct-1-en-3-one had been detected in walnuts as well as in walnut oil before,^6,37^ whereas HDMF had only been known in walnut oil,^38^ and *trans*-4,5-epoxy-(2*E*)-dec-2-enal and 2-methoxyphenol had previously been unknown as walnut and walnut oil constituents.

Looking at the compound classes, it became apparent that oxidation products of fatty acids constituted the major group within the 50 compounds listed in Table 1. This group included 16 aldehydes (**2**, **4**, **8**, **12**, **14**, **15**, **17**, **18**, **20**, **24**, **25**, **27**, **28**, **32**, **33**, and **38**), 3 ketones (**1**, **5**, and **7**), and 5 lactones (**34**, **36**, **43**, **45**, and **48**). Further compound classes were amino acid derivatives (**11**, **21**–**23**, **31**, **39**, **44**, **47**, **49**, and **50**), sugar-derived *O*-heterocycles (**29**, **37**, **40**–**42**, and **46**), *N*-heterocyclic pyrazines (**6**, **9**, and **13**), and terpenoids (**3**, **26**, and **35**).

### Odorant Quantitation and OAV Calculation

The 27 odorants which showed an FD factor of ≥16 in the screening (cf. Table 1) were selected for quantitation by GC–MS. Stable isotopically substituted odorants were used as internal standards (cf. Supporting Information, Table S2). For 23 compounds, isotopologues were available, allowing for an ideal compensation of potential workup losses. Only for compounds **20**, **23**, **27**, and **32**, no isotopologue was available. These compounds were quantitated using as internal standards the isotopologues of the isomeric compounds **24**, **22**, **28**, and **33**, respectively.

The results of the odorant quantitations showed concentrations between 0.0206 and 44,200 μg/kg, thus spanning a range of over 6 orders of magnitude (Table 2). High concentrations were determined for acetic acid (**10**; 44,200 μg/kg) and hexanoic acid (**30**; 2870 μg/kg), followed by (2*E*)-oct-2-enal (**8**; 439 μg/kg), butanoic acid (**19**; 184 μg/kg), (2*E*,4*E*)-deca-2,4-dienal (**28**; 178 μg/kg), (2*E*)-non-2-enal (**15**; 121 μg/kg), 3-methylbutanoic acid (**22**; 118 μg/kg), and vanillin (**50**; 105 μg/kg). The concentrations of (2*E*,4*E*,6*Z*)-nona-2,4,6-trienal (**32**) and sotolon (**46**), the compounds with the highest FD factors in the screening (cf. Table 1), were interestingly in the same range and amounted to 10.2 and 10.6 μg/kg, respectively. Situated on the low end were the concentrations of 3-*sec*-butyl-2-methoxypyrazine (**13**; <0.10 μg/kg), (5*Z*)-octa-1,5-dien-3-one (**7**; 0.0659 μg/kg), and 3-isopropyl-2-methoxypyrazine (**9**; 0.0206 μg/kg).

**Table 2:** Concentrations and OAVs of Important Odorants in Walnut Kernels

no.^a^	odorant	concentration in walnuts^b^ (μg/kg)	odor threshold concentration^c^ (μg/kg)	OAV^d^
10	acetic acid	44200	350	130
46	sotolon	10.6	0.23	46
27	(2*E*,4*Z*)-deca-2,4-dienal	46.7	2.8^e^	17
22	3-methylbutanoic acid	118	9.0	13
32	(2*E*,4*E*,6*Z*)-nona-2,4,6-trienal	10.2	1.1	9.3
30	hexanoic acid	2870	460	6.2
19	butanoic acid	184	34	5.4
38	*trans*-4,5-epoxy-(2*E*)-dec-2-enal	55.7	13	4.3
14	(2*Z*)-non-2-enal	13.6	3.6	3.8
8	(2*E*)-oct-2-enal	439	120	3.7
49	2-phenylacetic acid	90.2	26	3.5
28	(2*E*,4*E*)-deca-2,4-dienal	178	66	2.7
31	2-methoxyphenol	3.98	1.8	2.2
9	3-isopropyl-2-methoxypyrazine	0.0206	0.010	2.1
7	(5*Z*)-octa-1,5-dien-3-one	0.0659	0.044	1.5
24	(2*E*,4*E*)-nona-2,4-dienal	36.6	30	1.2
5	oct-1-en-3-one	7.42	6.9	1.1
15	(2*E*)-non-2-enal	121	140	<1
50	vanillin	105	140	<1
40	HDMF^f^	12.8	25	<1
23	2-methylbutanoic acid	52.6	110	<1
47	2′-aminoacetophenone	7.80	21	<1
13	3-*sec*-butyl-2-methoxypyrazine	<0.10	0.46	<1
20	(2*E*,4*Z*)-nona-2,4-dienal	3.48	16^e^	<1
17	(2*E*,6*Z*)-nona-2,6-dienal	8.76	65	<1
34	γ-octalactone	11.5	280	<1
12	(2*E*,4E)-hepta-2,4-dienal	13.3	710	<1

a Numbering according to Table 1.

b Mean of duplicates or triplicates; individual values and standard deviations are available in the Supporting Information, Table S3.

c Odor threshold concentrations determined in low odor sunflower oil.

d Odor activity value; calculated as a ratio of concentration to odor threshold concentration.

e Approximated from the odor threshold concentration of the (2*E*,4*E*)-isomer in low odor sunflower oil and the ratio of the odor threshold concentrations of the individual isomers in air (Supporting Information, Table S4).

f 4-Hydroxy-2,5-dimethylfuran-3(2*H*)-one.

By dividing the concentrations in the walnuts by the corresponding OTCs in oil, OAVs were calculated for the 27 odorants (Table 2). Among them, 17 odorants showed an OAV >1. The highest OAVs were calculated for vinegar-like smelling acetic acid (**10**; OAV 130), fenugreek-like smelling sotolon (**46**; OAV 46), fatty, deep-fried smelling (2*E*,4*Z*)-deca-2,4-dienal (**27**; OAV 17), sweaty, cheesy smelling 3-methylbutanoic acid (**22**; OAV 13), oatmeal-like smelling (2*E*,4*E*,6*Z*)-nona-2,4,6-trienal (**32**; OAV 9.3), and sweaty, cheesy smelling compounds hexanoic acid (**30**; OAV 6.2) and butanoic acid (**19**; OAV 5.4). Ten further odorants showed OAVs >1 but <5, including *trans*-4,5-epoxy-(2*E*)-dec-2-enal, (2*Z*)-non-2-enal, (2*E*)-oct-2-enal, 2-phenylacetic acid, (2*E*,4*E*)-deca-2,4-dienal, 2-methoxyphenol, 3-isopropyl-2-methoxypyrazine, (5*Z*)-octa-1,5-dien-3-one, (2*E*,4*E*)-nona-2,4-dienal, and oct-1-en-3-one.

OAV data are often used as the basis to discuss the relative contribution of individual odorants to the overall aroma. In fact, OAVs typically provide a much better approximation for the relative importance of odorants than FD factors resulting from AEDA because they are (1) not influenced by workup yields if based on proper quantitations, (2) consider the different volatility of the odorants because threshold data are obtained at room temperature and not at a hot sniffing-port, and (3) consider the different release behavior of the odorants because threshold data are determined in a matrix and not in air.^14^ However, the significance of OAV data depends largely on the similarity between the matrix used for the threshold determinations and the real food matrix. For foods high in water, OAVs based on OTCs determined in pure water are considered a good approximation. Considering that walnut kernels are low in water but high in fat, we employed OTCs determined in oil. However, this approach most probably led to an overestimation of the carboxylic acids. The OAVs determined for acetic acid (**10**; OAV 350), 3-methylbutanoic acid (**22**; OAV 13), hexanoic acid (**30**; OAV 6.2), and butanoic acid (**19**; OAV 5.4) were unrealistically high considering that in the natural matrix with some aqueous phase and a pH of 6.5, which is clearly beyond the pk_a_ values of the acids, the major parts would be deprotonated and therefore odor-inactive. Consequently, this would highlight the importance of the other compounds with high OAVs, namely, sotolon (**46**; OAV 46), (2*E*,4*Z*)-deca-2,4-dienal (**27**; OAV 17), and (2*E*,4*E*,6*Z*)-nona-2,4,6-trienal (**32**; OAV 9.3).

A second point that limits their significance is that OAVs do not consider interactions during the perception of odorant mixtures. Often, the odor of a mixture is dominated by some odorants while the odor of others is totally suppressed even though their OAVs are clearly >1.^14^ Sometimes, however, the combination of odorants generates a new synthetic odor that is not perceivable in the individual odorants. For example, it has been shown that the combination of cooked potato-like smelling methional and geranium leaf-like smelling (5*Z*)-octa-1,5-dien-3-one in a ratio of 100:1 results in a fishy smell.^39^ A similar effect might generate the characteristic aroma of walnuts. In order to elucidate the compounds being crucial for the characteristic walnut aroma, we proceeded with aroma reconstitution and omission experiments, for which we used a matrix that was closer to walnuts and apart from the predominating oil content additionally considered the water content and the pH of walnuts.

### Aroma Reconstitution and Omission Experiments

A first reconstitution model (Table 3, RM 1) included all 17 compounds for which OAVs ≥1 had been determined, dissolved in the natural concentrations (cf. Table 2) in a buffered oil/water emulsion. The second reconstitution model (Table 3, RM 2) was supposed to include only the five odorants with the highest OAVs of 9.3–130, while the other 12 compounds with rather low OAVs (1.1–6.2) should be omitted. However, we faced the problem that the (2*E*,4*Z*)-deca-2,4-dienal (**27**; OAV 17) reference contained some of the (2*E*,4*E*)-isomer (**28**; OAV 2.7). This prompted us—despite its low OAV—to additionally include (2*E*,4*E*)-deca-2,4-dienal in RM 2. When preparing the mixture, the amounts of the two reference compound samples were adjusted in order to result in the exact concentrations previously quantitated, thereby considering the amount of the (2*E*,4*E*)-isomer impurity in the (2*E*,4*Z*)-reference.

**Table 3:** Intensity of the Characteristic Walnut Note in Aroma Reconstitution Models based on 2 to 17 Odorants in Their Natural Concentrations

reconstitution model	odorants^a^	intensity “walnut”^b^
RM 1	all 17 odorants with OAVs >1	1.6
RM 2	**10**, **22**, **27**, **28**, **32**, **46**	2.1
RM 3	**10**, **22**	0.1
RM 4	**10**, **32**	0.3
RM 5	**10**, **27**/**28**	0.4
RM 6	**10**, **46**	1.0
RM 7	**22**, **27**/**28**	0.3
RM 8	**22**, **32**	0.4
RM 9	**22**, **46**	0.7
RM 10	**27**/**28**, **32**	0.5
RM 11	**27**/**28**, **46**	1.6
RM 12	**32**, **46**	2.3

a Odorant numbers according to Table 1.

b Assessors rated the intensity of the odor impression “walnut” on a scale from 0 to 3 with 0.5 increments and 0 = not perceptible, 1 = weak, 2 = moderate, and 3 = strong.

A trained sensory panel evaluated the two aroma reconstitution models RM 1 and RM 2 orthonasally in comparison to fresh walnut kernels. Assessors rated the intensity of the odor impression “walnut” on a scale from 0 to 3 with 0.5 increments and 0 = not perceptible, 1 = weak, 2 = moderate, and 3 = strong. To our surprise, model RM 2 with only 6 odorants was rated more walnut-like than model RM 1 with 17 odorants. Obviously, the odorants present in RM 1 but not in RM 2 reduced the typical walnut character in the overall odor profile. This prompted us to hypothesize that the compounds generating the characteristic walnut impression are among the six odorants included in RM 2. In the simplest case, a combination of two of the six would create a walnut aroma. Therefore, we aimed at proceeding with the sensory evaluation of binary mixtures. Given the impurity problem discussed before, the deca-2,4-dienal isomers **27** and **28** were treated as if they were just one compound, which was not considered a problem, because they showed virtually the same fatty, deep-fried odor. Results are displayed in Table 3 (RM 3–12).

A very characteristic walnut note was detected when oatmeal-like smelling (2*E*,4*E*,6*Z*)-nona-2,4,6-trienal (**32**) was combined with fenugreek-like smelling sotolon (**46**) (Table 3, RM 12). In this binary mixture, the intensity of the odor impression “walnut” was rated 2.3 out of 3. This score was clearly higher than the scores of all other mixtures including RM 2. Sotolon seems to contribute more to the walnut character than (2*E*,4*E*,6*Z*)-nona-2,4,6-trienal because all mixtures containing sotolon (RM 6, 9, 11, and 12) showed more walnut character (0.7–2.3) than the binary mixtures without sotolon (0.1–0.5). Actually, the term “walnut-like” has been used in some studies to describe the odor of sotolon.^40,41^ Nevertheless, in our experiments only the simultaneous presence of (2*E*,4*E*,6*Z*)-nona-2,4,6-trienal was able to push the fenugreek-like odor of sotolon toward a clear walnut character rated with the highest score of 2.3 (RM 12).^43−49^

### Sotolon and (2*E*,4*E*,6*Z*)-Nona-2,4,6-trienal in Other Tree Nuts

The aroma reconstitution and omission experiments detailed in the previous section suggested that a mixture of sotolon and (2*E*,4*E*,6*Z*)-nona-2,4,6-trienal in a ratio of ∼1:1 and at a concentration level of ∼10 μg/kg is crucial for the characteristic aroma of walnuts. In other tree nuts without a pronounced walnut character, the concentrations would most probably differ from the concentrations in the walnuts. To challenge this hypothesis, we additionally quantitated sotolon and (2*E*,4*E*,6*Z*)-nona-2,4,6-trienal in cashew nuts, hazelnuts, almonds, Brazil nuts, and pecan nuts.

Results of the quantitations (Table 4) revealed levels of (2*E*,4*E*,6*Z*)-nona-2,4,6-trienal below the OTC of 1.1 μg/kg (cf. Table 2) in cashew nuts, hazelnuts, and almonds. The sotolon concentration was also lower than in the walnuts and ranged from 2.15 to 3.55 μg/kg, thus still beyond its OTC. The sotolon/(2*E*,4*E*,6*Z*)-nona-2,4,6-trienal ratio was >5:1 and not ∼1:1 as in the walnuts. The Brazil nut sample was the only one in which the (2*E*,4*E*,6*Z*)-nona-2,4,6-trienal concentration was higher than the sotolon concentration resulting in a sotolon/(2*E*,4*E*,6*Z*)-nona-2,4,6-trienal ratio of 1:2.3, and again both concentrations were clearly lower than those in the walnuts. The lower amounts in combination with a sotolon/(2*E*,4*E*,6*Z*)-nona-2,4,6-trienal ratio clearly differing from 1:1 were in line with the lack of a walnut note in the cashew nut, hazelnut, almond, and Brazil nut samples. By contrast, the pecan nut sample showed some walnut character in the aroma, although not as pronounced as the walnuts. In view of their botany, this was not surprising because the pecan nut tree *Carya illinoinensis* and the walnut tree *J. regia* belong to the same family Juglandaceae. Actually, the (2*E*,4*E*,6*Z*)-nona-2,4,6-trienal concentration in the pecan nuts with 7.87 μg/kg was almost as high as in the walnuts, where 10.2 μg/kg had been determined and the sotolon concentration with 23.6 μg/kg was even higher, resulting in a ratio of sotolon to (2*E*,4*E*,6*Z*)-nona-2,4,6-trienal of 3:1. This raised the question which sotolon/(2*E*,4*E*,6*Z*)-nona-2,4,6-trienal ratio is actually the optimum to achieve the most characteristic walnut aroma. This question was addressed in the following experiments.

**Table 4:** Concentrations of (2*E*,4*E*,6*Z*)-Nona-2,4,6-trienal and Sotolon in Different Tree Nuts

		concentration (μg/kg)					
no.^a^	odorant	cashew nut^b^	hazelnut^b^	almond^b^	Brazil nut^b^	pecan nut^b^	walnut^c^
32	(2*E*,4*E*,6*Z*)-nona-2,4,6-trienal	<0.20	<0.20	0.560	1.18	7.87	10.2
46	sotolon	3.55	2.15	3.21	0.506	23.6	10.6

a Numbering according to Table 1.

b Mean of duplicates or triplicates; individual values and standard deviations are available in the Supporting Information, Table S5.

c Data taken from Table 2.

### Sensory Tests with Different Sotolon/(2*E*,4*E*,6*Z*)-Nona-2,4,6-trienal Mixtures

A first experiment was based on a 1:1 mixture of sotolon and (2*E*,4*E*,6*Z*)-nona-2,4,6-trienal at a concentration level of 10 μg/kg, thus approximating the situation in the walnuts. The matrix was the same oil/buffer mixture used for the reconstitution and omission tests. One of the two components was then reduced in its concentration to 3, 1 μg/kg, and finally omitted totally. This approach resulted in seven samples with different sotolon/(2*E*,4*E*,6*Z*)-nona-2,4,6-trienal ratios. The samples were presented to the trained sensory panel and assessors were asked to orthonasally rate the intensities of the three descriptors “walnut”, “fenugreek”, and “oatmeal”. The same scale previously used for the recombination and omission tests was used, which ranged from 0 to 3 with 0.5 increments and with 0 = not perceptible, 1 = weak, 2 = moderate, and 3 = strong.

**Figure 1:**
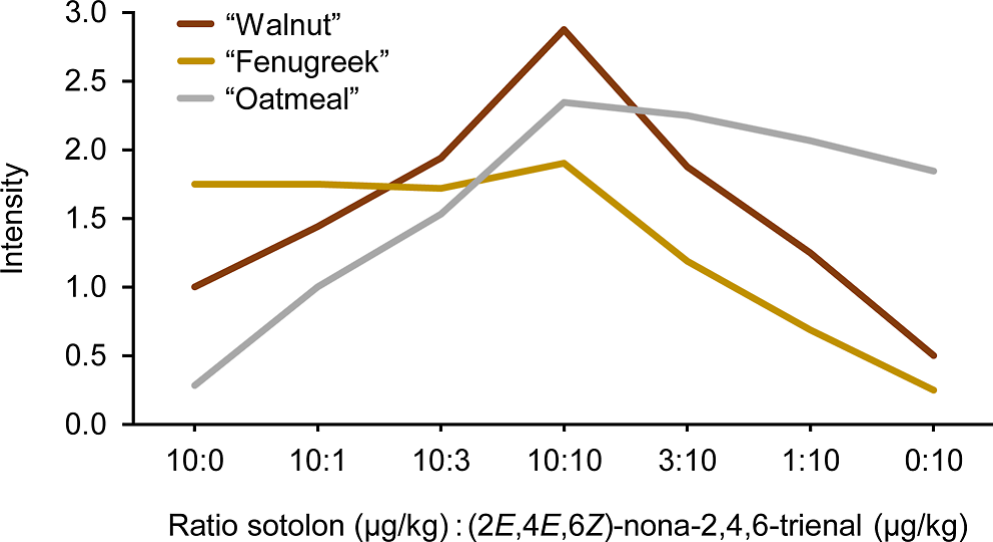
Impact of the ratio of sotolon to (2*E*,4*E*,6*Z*)-nona-2,4,6-trienal on the intensity of the odor impressions “walnut”, “fenugreek”, and “oatmeal” in model mixtures. Assessors employed a scale from 0 to 3 with 0.5 increments and 0 = not perceptible, 1 = weak, 2 = moderate, and 3 = strong.

The averaged results are depicted in Figure 1. The highest intensity of the walnut note was actually obtained when both compounds were present at 10 μg/kg, which were about the same concentrations as in the walnuts. Moderate intensity of the walnut note was still perceptible when one of the two compounds was present at 10 μg/kg and the other one at 3 μg/kg. However, when one of the two compounds was decreased to 1 μg/kg, the walnut character was only weak. The decrease in the walnut note was steeper when the sotolon concentration decreased, thus further confirming that sotolon contributes somewhat more to the walnut character than (2*E*,4*E*,6*Z*)-nona-2,4,6-trienal. It is noteworthy that, when sotolon and (2*E*,4*E*,6*Z*)-nona-2,4,6-trienal approached the 1:1 ratio and formed the walnut character, the original odor impressions of the two compounds did not vanish, but were still perceivable in parallel to the walnut note. In other words, the walnut note did not develop at the expense of the fenugreek-like note of sotolon and the oatmeal-like note of (2*E*,4*E*,6*Z*)-nona-2,4,6-trienal, but in addition.

**Figure 2:**
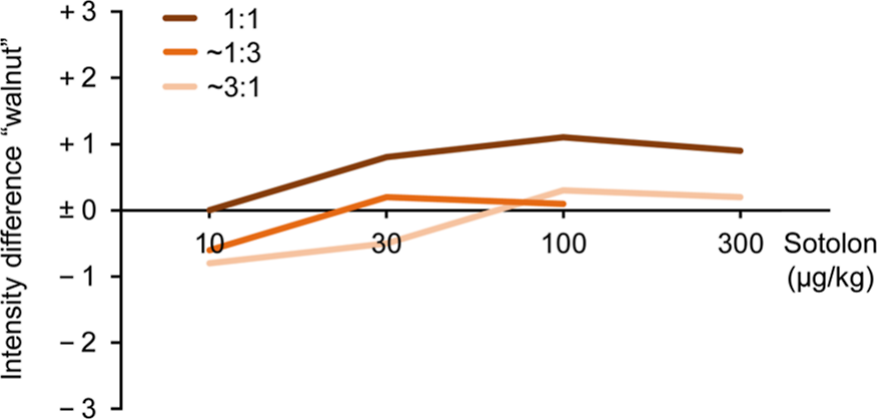
Change in the characteristic walnut note with increasing odorant concentrations (up to 300 μg/kg) shown for sotolon to (2*E*,4*E*,6*Z*)-nona-2,4,6-trienal ratios of 1:1, ∼1:3, and ∼3:1. Assessors rated the intensity difference on a scale from −3 to +3 with −3 = clearly weaker, −2 = moderately weaker, −1 = slightly weaker, 0 = no difference, +1 = slightly stronger, +2 = moderately stronger, and +3 = clearly stronger.

In a second experiment, we addressed the question of whether an increase of the sotolon and (2*E*,4*E*,6*Z*)-nona-2,4,6-trienal concentrations would be beneficial for the overall walnut aroma character or not. The concentration of the 1:1 mixture was increased from 10 μg/kg to 30, 100, and finally 300 μg/kg. To see if at higher overall concentrations the 1:1 mixtures would still represent the optimum ratio, we did the same with the mixtures in which the sotolon and (2*E*,4*E*,6*Z*)-nona-2,4,6-trienal concentrations differed by one step. All samples were evaluated against the mixture of both compounds at 10 μg/kg and the assessors were asked to rate the difference in the intensity of the walnut note on a scale from −3 to +3 with −3 = clearly weaker, −2 = moderately weaker, −1 = slightly weaker, 0 = no difference, +1 = slightly stronger, +2 = moderately stronger, and +3 = clearly stronger. Averaged results (Figure 2) clearly showed that also at higher overall concentrations, the 1:1 ratio of sotolon and (2*E*,4*E*,6*Z*)-nona-2,4,6-trienal resulted in the highest rating for the walnut note. The walnut note of a 1:1 mixture of sotolon and (2*E*,4*E*,6*Z*)-nona-2,4,6-trienal intensified when the concentrations increased from 10 to 30 μg/kg and from 30 to 100 μg/kg but showed a slight decrease when the concentrations further increased from 100 to 300 μg/kg. In conclusion, the sensory tests with the different sotolon/(2*E*,4*E*,6*Z*)-nona-2,4,6-trienal mixtures suggested that a 1:1 mixture of both compounds at a concentration level of 100 μg/kg is desirable to achieve an intense walnut-like aroma character. This result may be helpful to evaluate the aroma of different walnut varieties on an analytical basis and to set targets for the breeding of new walnut cultivars.

**Figure 3:**
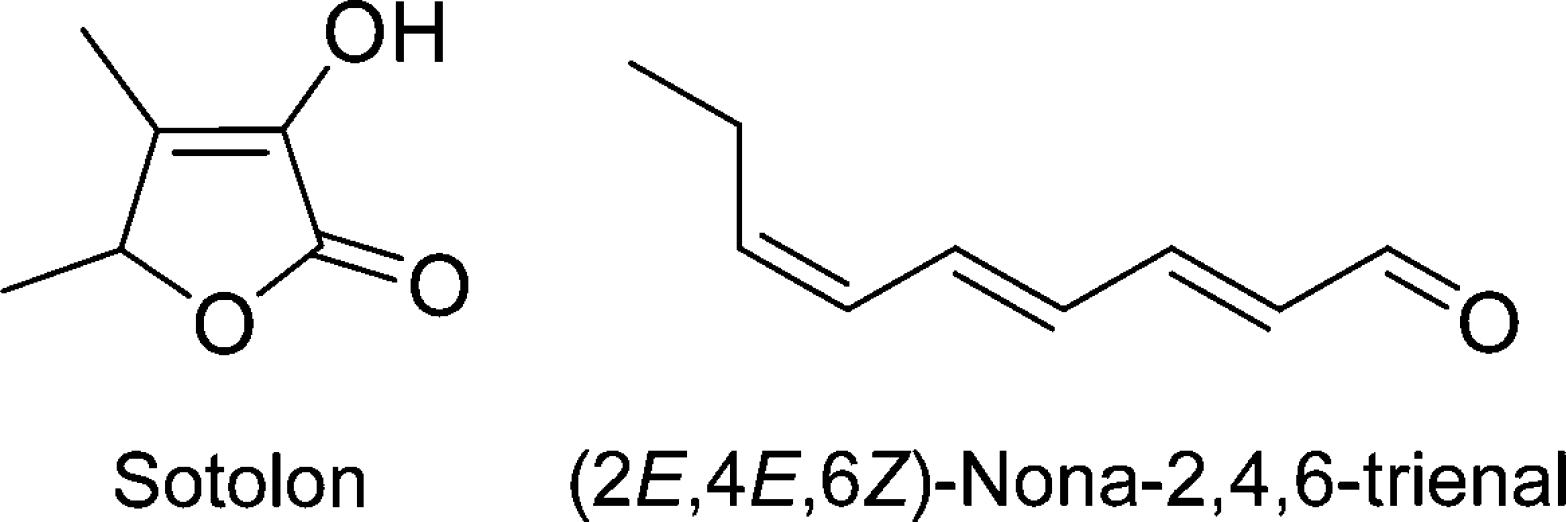
Key odorants in walnuts.

In summary, our study showed that the compounds responsible for the characteristic aroma of unprocessed walnuts are fenugreek-like smelling sotolon and oatmeal-like smelling (2*E*,4*E*,6*Z*)-nona-2,4,6-trienal (Figure 3). It was surprising that both compounds had not been detected in walnuts before, although molecular sensory science approaches had been applied in previous studies. It is somewhat speculative to discuss possible reasons for that. However, representativeness of the walnut sample in terms of the aroma properties, sample pretreatment before extraction—particularly the degree of crushing, enzyme inhibition, and water addition, artifact-avoiding isolation of the volatile fraction, and the experience of the assessors performing GC–O may have been critical points. Our results also nicely illustrate that it is not feasible to define key odorants on the basis of OAVs as suggested by Liu et al.^11^ OAVs provide a useful tool to select the compounds for the subsequent reconstitution and omission tests but do not allow unequivocally assessing the importance of individual compounds for the overall aroma. An aroma reconstitution experiment is essential to confirm that all key odorants have been captured and only if the reconstitution experiment was successful, omission tests finally allow to identify the key odorants.^14^

In the future, the targeted quantitation of sotolon and (2*E*,4*E*,6*Z*)-nona-2,4,6-trienal may not only be useful for quality control but can also be included in studies aimed at a deeper molecular understanding of variety selection, agricultural parameters, post-harvest handling, and processing on the sensory characteristics of walnuts and walnut products.

## Supporting Information Available

Information on GC instruments; references on synthetic procedures to isotopically substituted odorants; stable isotopically substituted internal standards, quantifier ions, and calibration lines used in the quantitation assays; individual concentration data used for mean calculations and standard deviations; and odor threshold concentrations of **20**, **24**, **27**, and **28** in air (PDF)

## Abbreviations

AEDA: aroma extract dilution analysis
aSAFE: automated solvent-assisted flavor evaporation
EHMF: 2-ethyl-4-hydroxy-5-methylfuran-3-one
FD: flavor dilution
GC: gas chromatography
GC–GC–HRMS: gas chromatography–gas chromatography–high-resolution mass spectrometry
GC–MS: gas chromatography–mass spectrometry
GC–O: gas chromatography–olfactometry
GC–O/FID: gas chromatography–olfactometry/flame ionization detector
HDMF: 4-hydroxy-2,5-dimethylfuran-3(2*H*)-one
OAV: odor activity value
OTC: odor threshold concentration
RM: reconstitution model
RI: retention index
SAFE: solvent-assisted flavor evaporation

## Nomenclature

2′-aminoacetophenone: 1-(2-aminophenyl)ethan-1-one
cyclotene: 2-hydroxy-3-methyl-2-cyclopenten-1-one
(2*Z*,4*Z*)-δ-deca-2,4-dienolactone: 6-pentylpyran-2-one
γ-decalactone: 5-hexyloxolan-2-one
(6*Z*)-γ-dodec-6-enolactone: 5-[(2*Z*)-oct-2-enyl]oxolan-2-one
eugenol: 2-methoxy-4-(prop-2-en-1-yl)phenol
α-farnesene: (3*E*,6*E*)-3,7,11-trimethyldodeca-1,3,6,10-tetraene
β-ionone: (3*E*)-4-(2,6,6-trimethylcyclohex-1-en-1-yl)but-3-en-2-one
3-isopropyl-2-methoxypyrazine: 2-methoxy-3-(propan-2-yl)pyrazine
maltol: 3-hydroxy-2-methyl-4*H*-pyran-4-one
methional: 3-(methylsulfanyl)propanal
γ-octalactone: 5-butyldihydro-2(3*H*)-furanone
δ-octalactone: 6-butyldihydro-2(3*H*)-furanone
3-*sec*-butyl-2-methoxypyrazine: 2-(butan-2-yl)-3-methoxypyrazine
γ-terpinene: 1-methyl-4-propan-2-ylcyclohexa-1,4-diene
sotolon: 3-hydroxy-4,5-dimethylfuran-2(5*H*)-one
*trans*-4,5-epoxy-(2*E*)-dec-2-enal: (2*E*)-3-[(3-(2*R*,3*R*) and/or (2*S*,3*S*)-pentyloxiran-2-yl]prop-2-enal
vanillin: 4-hydroxy-3-methoxybenzaldehyde
