# Supplementary material for: Sotolon and (2E,4E,6Z)-Nona-2,4,6-trienal Are the Key Compounds in the Aroma of Walnuts
Source: J Agric Food Chem. 2023 Apr 26;71(18):7099–108. doi: 10.1021/acs.jafc.3c01002 (PMC10176575; doi:10.1021/acs.jafc.3c01002)
Supplement: Supplementary file 1 — jf3c01002_si_001.pdf [file jf3c01002_si_001.pdf]

Supporting Information

Sotolon and (2*E*,4*E*,6*Z*)-Nona-2,4,6-trienal Are the Key Compounds in the Aroma of Walnuts

Christine A. Stübner and Martin Steinhaus*

Leibniz Institute for Food Systems Biology at the Technical University of Munich (Leibniz-LSB@TUM), Lise-Meitner-Straße 34, 85354 Freising, Germany

*E-mail: martin.steinhaus@tum.de

Overview

Additional Information on GC Instruments

GC–O/FID Instrument

GC–MS Instrument

Heart-Cut GC–GC–MS Instrument

Heart-Cut GC–GC–HRMS Instrument

Comprehensive Two-Dimensional GC×GC–MS Instrument

Additional Tables

Table S1. References on Synthetic Procedures to Isotopically Substituted Odorants

Table S2. Stable Isotopically Substituted Internal Standards, Quantifier Ions, and Calibration Lines Used in the Quantitation Assays

Table S3. Concentrations of Important Odorants in Walnut Kernels

Table S4. Odor Threshold Concentrations of 20, 24, 27, and 28 in Air

Table S5. Concentrations of (2*E*,4*E*,6*Z*)-Nona-2,4,6-trienal (32) and Sotolon (46) in Other Tree Nuts

GC–O/FID Instrument

A Trace Gas Chromatograph Ultra (Thermo Fisher Scientific; Dreieich, Germany) was equipped with a cold on-column injector, a flame ionization detector (FID), and a sniffing-port custom-made from aluminum as detailed in *J. Agric. Food Chem*. **2008**, *56*, 4120–4127. The column was either a DB-FFAP column, 30 m × 0.32 mm i.d., 0.25 µm film thickness, or a DB-5 column, 30 m × 0.25 mm i.d., 0.25 µm film thickness, both purchased from Agilent (Waldbronn, Germany). The carrier gas was helium at 57 kPa constant pressure (DB-FFAP) or 78 kPa constant pressure (DB-5). The injection volume was 1 µL. The initial oven temperature of 40 °C was held for 2 min, followed by a gradient of 6 °C/min until a final temperature of 230 °C (DB-FFAP) or 240 °C (DB-5), which was held for 5 min. A Y-shaped glass splitter connected the end of the column with two uncoated but deactivated fused silica capillaries, each 50 cm × 0.25 mm i.d., which delivered the column effluent in two equal parts to the FID (250 °C base temperature) and the sniffing port (230 °C base temperature), respectively. During a GC−O run, the FID signal was recorded by a computer using the Olfactory Voicegram software V.2.2.17 (GL Sciences; Eindhoven, Netherlands). The assessor evaluated the odor of the effluent with the nose placed directly above the sniffing port. Whenever he perceived an odor, he selected an appropriate odor description from an adjustable palette by mouse click. The software included the description with the retention time in the chromatogram. Retention indices were calculated from the retention times of the odor-active compounds and the retention times of adjacent *n*-alkanes by linear interpolation as detailed in *J. Chromatogr. A*. **1963**, *11*, 463–471.

GC–MS Instrument

A 7890B gas chromatograph (Agilent) was equipped with a Varian PAL Autosampler (Varian; Darmstadt, Germany) and a cold on-column injector. The column was either a DB-FFAP, 30 m × 0.25 mm i.d., 0.25 µm film thickness or a DB-5 column, 30 m × 0.25 mm i.d., 0.25 µm film thickness, both purchased from Agilent. The carrier gas was helium at 1.0 mL/min constant flow. The injection volume was 1 µL. The oven temperature programs were identical to those used in the GC–O/FID analyses. The GC was connected to a 220 ion trap mass spectrometer (Varian) used in the chemical ionization (CI) mode with methanol as reagent gas and a scan range of *m/z* 60–250. Data were analyzed with the MS Workstation software, version 7.0.2 (Agilent).

Heart-Cut GC–GC–MS Instrument

A Trace GC Ultra (Thermo Fisher Scientific) was equipped with a Combi PAL autosampler (CTC Analytics; Zwingen, Switzerland), a cold on-column injector, an FID (250 °C base temperature), and a custom-made sniffing port (cf. *J. Agric. Food Chem*. **2008**, *56*, 4120–4127; 230 °C base temperature). The column was a DB-FFAP column, 30 m × 0.32 mm i.d., 0.25 µm film thickness (Agilent). The carrier gas was helium at 110 kPa constant pressure. The injection volume was 2 µL. The initial oven temperature of 40 °C was held for 2 min, followed by gradients of 6–40 °C/min until a final temperature of 230 °C, which was held for 5 min. The end of the column was connected to a moving column stream switching (MCSS) system (Thermo Fisher Scientific) used for heart-cutting. Helium at 50 kPa was used as make-up gas. The MCSS system directed the eluate of the column via uncoated but deactivated fused silica capillaries (0.32 mm i.d.) time-programmed either simultaneously to the FID and the sniffing port used as monitor detectors or to a second GC column, which was a DB-1701 column, 30 m × 0.25 mm i.d., 0.25 µm film thickness (Agilent). This column was installed in a separate gas chromatograph, which was a CP 3800 GC (Varian). The capillary to the second column first passed through a heated (250 °C) hose connecting the two gas chromatographs and then through a liquid nitrogen-cooled trap installed inside the oven of the second gas chromatograph and used to refocus the heart-cut. The initial temperature of the second oven was 40 °C and was held for 2 min, followed by gradients of 6–30 °C/min to a final temperature of 240 °C. The end of the second column was connected to a Saturn 2200 mass spectrometer (Varian) used in CI mode with methanol as reagent gas and a scan range of *m/z* 60–250. Data were analyzed with the MS Workstation software, version 6.9.3 (Varian).

Heart-Cut GC–GC–HRMS Instrument

A Trace 1310 gas chromatograph (Thermo Fisher Scientific) was equipped with a TriPlus RSH autosampler, a programmed temperature vaporizing (PTV) injector, an FID (250 °C base temperature), and a custom-made sniffing port (cf. *J. Agric. Food Chem*. **2008**, *56*, 4120–4127; 230 °C base temperature). The column was a DB-FFAP column, 30 m × 0.25 mm i.d., 0.25 µm film thickness (Agilent). The carrier gas was helium at 1 mL/min constant flow. The injection volume was 1 µL. The initial oven temperature of 40 °C was held for 2 min, followed by a gradient of 6 °C/min until a final temperature of 230 °C, which was held for 5 min. The end of the column was connected to a Deans switch (Trajan; Ringwood, Australia) used for heart-cutting. The Deans switch directed the eluate of the column via uncoated but deactivated fused silica capillaries (0.1 mm i.d.) time-programmed either simultaneously to the FID and the sniffing port used as monitor detectors or to a second GC column, which was a DB-1701 column, 30 m × 0.25 mm i.d., 0.25 µm film thickness (Agilent). This column was installed in a second Trace 1310 gas chromatograph. The capillary to the second column first passed through a heated (250 °C) hose connecting the two gas chromatographs and then through a liquid nitrogen-cooled trap used to refocus the heart-cut. The initial temperature of the second oven was 40 °C and was held for 2 min, followed by a gradient of 6 °C/min to a final temperature of 240 °C, which was held for 5 min. The end of the second column was connected to a Q Exactive GC orbitrap mass spectrometer (Thermo Fisher Scientific) operated in high-resolution mode with negative CI or positive CI using isobutane as reagent gas and a scan range of *m/z* 90−150. Data were analyzed with the Xcalibur software (Thermo Fisher Scientific).

Comprehensive Two-Dimensional GC×GC–MS Instrument

A 6890 gas chromatograph (Agilent) was equipped with a GC PAL autosampler (CTC Analytics) and a CIS 4 injector (Gerstel, Mülheim an der Ruhr, Germany). The column in the first dimension was a DB-FFAP column, 30 m × 0.25 mm i.d., 0.25 µm film thickness (Agilent). The carrier gas was helium at 2 mL/min constant flow. The injection volume was 2 µL. The initial oven temperature of 40 °C was held for 2 min, followed by a gradient of 6 °C/min until a final temperature of 230 °C, which was held for 5 min. The end of the first column was connected via a liquid nitrogen-cooled dual-stage quad-jet modulator (Leco; Mönchengladbach, Germany) to a DB-5 column, 3 m × 0.15 mm i.d., 0.3 µm film thickness (Agilent) inside the secondary oven, which was mounted inside the primary GC oven. The modulation time was 4 s. The initial temperature of the second oven of 80 °C was held for 2 min, followed by a gradient of 6 °C/min until a final temperature of 250 °C, which was held for 5 min. The end of the second column was connected to Pegasus II TOF mass spectrometer (Leco) operated in the electron ionization (EI) mode at 70 eV with a scan range of *m*/*z* 35-350 and a scan rate of 100 spectra/s. Data were analyzed with the ChromSpace software (SepSolve Analytical, Peterborough, UK).

Table S1. References on Synthetic Procedures to Isotopically Substituted Odorants

compound	Reference
($^{2}H_2$)-**5**	*Indoor Air* **2021**, *31*, 1038–1049.
($^{2}H_2$)-**7**	*J. Agric. Food Chem*. **2018**, *66*, 1452–1460.
($^{2}H_2$)-**8**	*J. Agric. Food Chem.* **2020**, *68*, 15284–15291.
($^{13}C_2$)-**12**	Thorkildsen, J. Characterization of key aroma compounds formed during peroxidation of bulk fish oil and insights into oxidation mechanisms. Deutsche Forschungsanstalt für Lebensmittelchemie: Freising, Germany, 2014.
($^{2}H_3$)-**13**	*Flavour Fragrance J.* **1998**, *13*, 115–124.
($^{2}H_2$)-**14**	*Indoor Air* **2021**, *31*, 1038–1049.
($^{2}H_2$)-**15**	*J. Agric. Food Chem.* **2019**, *67*, 5838–5846.
($^{2}H_2$)-**17**	*Lebensm.-Wiss. Technol.* **1990**, *23*, 513–522.
($^{2}H_2$)-**19**	*Indoor Air* **2021**, *31*, 1038–1049.
($^{2}H_2$)-**22**	*J. Agric. Food Chem.* **2005**, *53*, 6049–6055.
($^{13}C_2$)-**24**	*Indoor Air* **2021**, *31*, 1038–1049.
($^{13}C_2$)-**28**	*Eur. Food Res. Technol*. **2021**, *247*, 1263–1275.
($^{2}H_3$)-**31**	*Z. Lebensm.-Unters. Forsch.* **1993**, *196*, 417–422.
($^{13}C_2$)-**33**	*J. Agric. Food Chem.* **2005**, *53*, 8699–8705.
($^{2}H_2$)-**34**	*J. Agric. Food Chem.* **2016**, *64*, 8168–8178.
($^{2}H_2$)-**38**	Lipids **1999**, *34*, 1117–1126.
($^{13}C_2$)-**40**	*J. Agric. Food Chem.* **1997**, *45*, 2642–2648.
($^{13}C_2$)-**46**	Blank, I. et al. In *Progress in Flavour Precursor Studies*; Schreier, P., Winterhalter, P., Eds.; Allured Publishing: Carol Stream, IL, 1993; pp. 103–109.
($^{2}H_3$)-**47**	*J. AOAC Int.* **1996**, *79*, 583–586.
($^{2}H_3$)-**50**	*Flavour Fragrance J.* **1995**, *10*, 1–7.

Table S2. Stable Isotopically Substituted Internal Standards, Quantifier Ions, and Calibration Lines Used in the Quantitation Assays

odorant	internal standard	quantifier ions (*m*/*z*)		calibration line equation	R^2
		analyte	standard		
**5**	($^2H_{2-4}$)-**5**	127	129–131	y = 1.035x − 0.084	0.999
**7**	($^2H_2$)-**7**	109	111	y = 0.866x + 0.009	1.000
**8**	($^2H_2$)-**8**	127	129	y = 0.853x − 0.059	0.999
**9**	($^2H_3$)-**9**	137	140	y = 0.651x + 0.146	0.992
**10**	($^{13}C_2$)-**10**	60	62	y = 1.025x − 0.007	0.999
**12**	($^{13}C_2$)-**12**	110	112	y = 0.853x − 0.059	0.999
**13**	($^2H_3$)-**13**	138	141	y = 1.105x − 0.189	0.993
**14**	($^2H_2$)-**14**	123	125	y = 1.071x − 0.066	0.993
**15**	($^2H_2$)-**15**	141	143	y = 1.117x − 0.003	0.999
**17**	($^2H_2$)-**17**	139	141	y = 0.614x + 0.086	0.999
**19**	($^2H_2$)-**19**	89	91	y = 0.970x + 0.029	0.998
**20**[a]	($^{13}C_2$)-**24**	138	140	y = 0.843x + 0.065	0.999
**22,23**[b]	($^2H_2$)-**22**	85	87	y = 0.946x + 0.053	0.991
**24**	($^{13}C_2$)-**24**	138	140	y = 0.843x + 0.065	0.999
**27**[c]	($^{13}C_2$)-**28**	81	83	y = 0.981x + 0.159	0.991
**28**	($^{13}C_2$)-**28**	81	83	y = 0.981x + 0.159	0.991
**30**	($^2H_3$)-**30**	99	102	y = 0.923x − 0.006	0.999
**31**	($^2H_3$)-**31**	124	127	y = 0.882x + 0.075	0.999
**32**	($^{13}C_2$)-**33**	136	138	y = 1.005x − 0.098	1.000
**34**	($^2H_2$)-**34**	143	145	y = 1.097x − 0.214	0.993
**38**	($^2H_2$)-**38**	97	99	y = 0.913x + 0.114	0.998
**40**	($^{13}C_2$)-**40**	128	130	y = 0.849x + 0.089	0.997
**46**	($^{13}C_2$)-**46**	128	130	y = 1.036x − 0.151	0.999
**47**	($^2H_3$)-**47**	135	138	y = 1.030x − 0.071	0.998
**49**	($^{13}C_2$)-**49**	136	138	y = 0.907x + 0.054	0.999
**50**	($^2H_3$)-**50**	151	154	y = 1.138x − 0.075	0.999

[a]**20** was quantitated using the calibration line determined for **24**. [b]**22** and **23** were not separated on the GC column used; they were quantitated as a sum and individual concentrations were subsequently calculated from the result and the ratio of **22** to **23** obtained from the intensities of *m*/*z* 60 and *m*/*z* 74 of the respective peak after GC-MS(EI) as detailed in *J. Agric. Food Chem.* **2008**, *56*, 4120–4127. [c]**27** was quantitated using the calibration line determined for **28**.

Table S3 Concentrations of Important Odorants in Walnut Kernels

odorant	concentration (µg/kg)			
	experiment 1	experiment 2	experiment 3	mean ± SD (CV)[a]
**5**	7.76	7.07		7.42 ± 0.49 (7%)
**7**	0.0586	0.0702	0.0689	0.0659 ± 0.0064 (10%)
**8**	313	528	476	439 ± 113 (26%)
**9**	0.0228	0.0207	0.0282	0.0206 ± 0.0023 (11%)
**10**	43900	41700	47000	44200 ± 2700 (6%)
**12**	13.9	12.6	13.3	13.3 ± 0.7 (5%)
**13**	≤0.0100[b]	≤0.0303[b]	≤0.0166[b]	
**14**	12.7	14.0	14.3	13.6 ± 0.9 (6%)
**15**	114	105	145	121 ± 21 (17%)
**17**	7.37	7.17	11.7	8.76 ± 3.59 (30%)
**19**	222	169	161	184 ± 33 (18%)
**20**	3.30	4.26	2.87	3.48 ± 0.71 (20%)
**22**	93.0	139	123	118 ± 23 (19%)
**23**	41.4	61.7	54.6	52.6 ± 10.3 (20%)
**24**	32.7	44.6	35.5	36.6 ± 6.9 (19%)
**27**	49.6	43.7		46.7 ± 4.1 (9%)
**28**	175	211	147	178 ± 32 (18%)
**30**	3490	2490	2620	2870 ± 550 (19%)
**31**	3.79	3.83	4.31	3.98 ± 0.29 (7%)
**32**	9.93	9.00	11.8	10.2 ± 1.4 (14%)
**34**	10.8	12.3	11.2	11.5 ± 0.8 (7%)
**38**	72.9	45.1	49.2	55.7 ± 15 (27%)
**40**	11.5	15.0	12.0	12.8 ± 1.9 (15%)
**46**	8.66	11.3	11.9	10.6 ± 1.73 (16%)
**47**	7.60	8.35	7.46	7.80 ± 0.48 (6%)
**49**	106	87.0	77.4	90.2 ± 14.5 (16%)
**50**	107	99.2	109	105 ± 5 (5%)

[a]SD, standard deviation; CV, coefficient of variation. [b]No analyte peak was observed; values were derived from the integration of the background noise.

Table S4. Odor Threshold Concentrations of 20, 24, 27, and 28 in Air[a]

odorant	conc. (µg/mL)	FD factor	conc. (E2D) (µg/mL)	FD factor (E2D)	OTC in air (ng/L)
**20**	39.4	1024	29.2	64	0.23
**24**	36.8	512	29.2	64	0.43
**27**	5.70	256	4.38	8	0.11
**28**	20.8	128	21.9	128	2.6

[a]Threshold concentrations were determined using the approach detailed in *Z. Lebensm.-Unters. Forsch.* **1987**, *184*, 277–282, and a threshold concentration of 2.7 ng/L for the internal standard (2*E*)-dec-2-enal (E2D) taken from *Ann. N.Y. Acad. Sci.* **1974**, *237*, 209–216.

Table S5. Concentrations of (2*E*,4*E*,6*Z*)-Nona-2,4,6-trienal (32) and Sotolon (46) in Other Tree Nuts

tree nut	odorant	concentration (µg/kg)			
		experiment 1	experiment 2	experiment 3	mean ± SD (CV)[a]
cashew nut	**32**	≤0.0959[b]	≤0.0954[b]	≤0.119[b]	
	**46**	3.71	3.42	3.51	3.55 ± 0.15 (4%)
hazelnut	**32**	≤0.191[b]	≤0.105[b]	≤0.171[b]	
	**46**	2.65	1.88	1.92	2.15 ± 0.44 (20%)
almond	**32**	0.644	0.550	0.486	0.560 ± 0.080 (14%)
	**46**	3.48	3.31	2.83	3.21 ± 0.03 (10%)
Brazil nut	**32**	1.27	1.09		1.18 ± 0.13 (11%)
	**46**	0.495	0.481	0.542	0.506 ± 0.032 (6%)
pecan nut	**32**	7.46	8.28		7.87 ± 0.58 (7%)
	**46**	25.1	22.6	23.1	23.6 ± 1.3 (5%)

[a]SD, standard deviation; CV, coefficient of variation. [b]No analyte peak was observed; values were derived from the integration of the background noise.
